# Fish associations with shallow water subsea pipelines compared to surrounding reef and soft sediment habitats

**DOI:** 10.1038/s41598-021-85396-y

**Published:** 2021-03-18

**Authors:** Karl D. Schramm, Michael J. Marnane, Travis S. Elsdon, Christopher M. Jones, Benjamin J. Saunders, Stephen J. Newman, Euan S. Harvey

**Affiliations:** 1 School of Molecular and Life Sciences, Curtin University, Bentley, WA 6102 Australia; 2 Chevron Technical Center, 250 St Georges Tce, Perth, WA 6000 Australia; 3 Chevron Australia Pty Ltd, 250 St Georges Tce, Perth, WA 6000 Australia; 4 Western Australian Fisheries and Marine Research Laboratories, Department of Primary Industries and Regional Development, Government of Western Australia, P.O. Box 20, North Beach, WA 6920 Australia

**Keywords:** Marine biology, Biodiversity

## Abstract

Offshore decommissioning activities are expected to increase as oil and gas subsea infrastructure becomes obsolete. Decisions on decommissioning alternatives will benefit from quantifying and understanding the marine communities associated with these structures. As a case study, fish assemblages associated with an inshore network of subsea pipelines located on the North West shelf of Western Australia were compared to those in surrounding natural reef and soft sediment habitats using remotely operated vehicles fitted with a stereo-video system (stereo-ROVs). The number of species, the abundance, biomass, feeding guild composition and the economic value of fishes were compared among habitats. The community composition of fish associated with pipelines was distinct from those associated with natural habitats, and was characterised by a greater abundance and/or biomass of fish from higher trophic levels (e.g. piscivores, generalist carnivores and invertivores), including many species considered to be of value to commercial and recreational fishers. Biomass of fish on pipelines was, on average, 20 times greater than soft sediments, and was similar to natural reefs. However, the biomass of species considered important to fisheries recorded on the pipelines was, on average 3.5 times greater than reef and 44.5 times greater than soft sediment habitats. This study demonstrates that fish assemblages on the pipeline infrastructure exhibit high ecological and socioeconomic values.

## Introduction

There are approximately 7500 oil and gas structures in the marine environment globally, many of which will require decommissioning in the near future as they reach the end of their production life^1,2^. In most countries, current decommissioning policies require the complete removal of infrastructure. This is in alignment with international obligations (i.e. the United Nations Convention on the Law of the Sea (UNCLOS) and the Convention on the Prevention of Marine Pollution by Dumping of Wastes and Other Matter (London Convention)^3,4^). In Australia, the default policy position on decommissioning is complete removal^5^. However, with growing evidence that oil and gas structures have the potential to function as artificial reefs (e.g.^6–12^), there is a compelling environmental case for consideration of in situ decommissioning alternatives. These alternatives include leaving the infrastructure in place, partial removal, toppling onto the seafloor or relocating to a designated reefing site^3,13,14^. Alternative decommissioning strategies could be supported if there is information demonstrating equal or better environmental and safety outcomes in comparison to complete removal^15^.

There is demonstrated evidence that oil and gas infrastructure can support a high abundance and diversity of sessile invertebrates and fish, including species that are considered commercially and recreationally important^3,9^, and/or are of high conservation value^10,16^. The option of leaving these structures in the water may potentially benefit fisheries through increased catch^17^. They may also provide opportunities for diver-based tourism where structures are readily accessible^18^. Some of these structures have also been documented as having potential conservation benefits^10,19,20^. However, there are concerns that the aggregation of fish may also lead to overfishing and depletion of fish stocks, especially if attraction is driving these associations^21–24^, although see^7^. Additionally, it has been suggested that offshore infrastructure may facilitate the propagation of invasive species by providing a mechanism for connecting habitat mosaics^25,26^. Other considerations often associated with *in-situ* decommissioning alternatives include potential leaching of contaminants, snagging risk for trawl fisheries and shipping navigational hazards^27,28^. From an ecological perspective, it is important that rigorous scientific data is collected to characterise fish assemblages associated with these structures in order to weigh the environmental, social and economic value of retaining these habitats against other benefits and risks.

Subsea pipelines are an integral component of oil and gas operations and form extensive networks on the seafloor. Despite their prevalence in our oceans there are few environmental studies that assess the ecological role of subsea pipelines as habitat (although see^29–34^), with the majority of literature focused on oil and gas platforms^14^. Research is now beginning to demonstrate the potential role subsea pipelines may serve in the marine environment. For example, a colony of Australian fur seals (*Arctocephalus pusillus doriferus*) in the Bass Strait, south-eastern Australia, has been documented using subsea pipelines to search for prey^35^. Similarly, fish have been documented utilising subsea pipelines as habitat, with Love and York^34^ reporting that fish densities were six to seven times greater on pipelines compared to the adjacent seafloor in the Santa Barbara Channel, Southern California. Studies specific to north Western Australia^29,32,33^ have also documented a high diversity and abundance of fish on pipelines, including species that are considered important to fisheries (e.g. lutjanids (snappers) and epinephelids (groupers)). Higher distributions of fish were observed near spanning pipelines (i.e. unsupported pipe where seabed sediment has been removed by water flow scouring), suggesting that these structures may be favourable places for refuge and access to food (e.g. ambush behaviours) for some species^29,32,33^.

Studies that have assessed fish associations with subsea pipelines have either used existing industry remotely operated vehicle (ROV) video footage^31–33^, small submersibles^34^, or Baited Remote Underwater stereo-Video systems (stereo-BRUVs)^29,30^ as a means of sampling. The use of a mini-ROV fitted with a stereo-video system (stereo-ROV) may be a more appropriate sampling approach for assessing fish associations on pipelines, as the stereo camera setup provides per unit area measurements of fish and accurate length data for biomass estimates in situ^12^. Furthermore, the majority of studies have assessed pipelines in relatively deep waters, ranging from 56 to 230 m (although see^30^, where fish surveys started at 15 m depth), and where surrounding habitat consisted predominantly of soft sediment. There is little information describing how fish communities interact with subsea pipelines at shallow depths (< 30 m), particularly where surrounding habitats are complex, such as coral reefs.

The North West Shelf of Western Australia encompasses the Northern Carnarvon and Roebuck Basins, where several thousand kilometres of subsea pipeline exist across multiple oil and gas projects^36^. These structures are predominantly situated over soft sediment habitat and sparse, sponge garden communities. However, at shallower depths (< 30 m) they also lie within, or adjacent to complex reef systems, which include communities of hard and soft corals. Studies in this region have shown the inshore fish assemblage to be highly diverse and include species which are endemic (e.g. *Lethrinus punctulatus*), protected (e.g. *Epinephelus lanceolatus*), and of importance to commercial and recreational fishers^37–40^.

With decommissioning activities expected to increase in the future, understanding the ecological roles of oil and gas structures, including subsea pipelines, will contribute valuable information to the decision-making process on decommissioning alternatives. This study aims to compare fish assemblages on exposed shallow-water subsea pipelines to those observed in nearby natural reef and soft sediment habitats using stereo-ROVs. We assessed the number of species, abundance, biomass, feeding guild composition and the potential economic value of fish communities associated with pipelines near Thevenard Island, Western Australia. We also surveyed nearby natural reef and soft sediment habitats in order to contextualise the value of pipelines as habitat for shallow-water fish communities.

## Methods

### Study area

**Figure 1:**
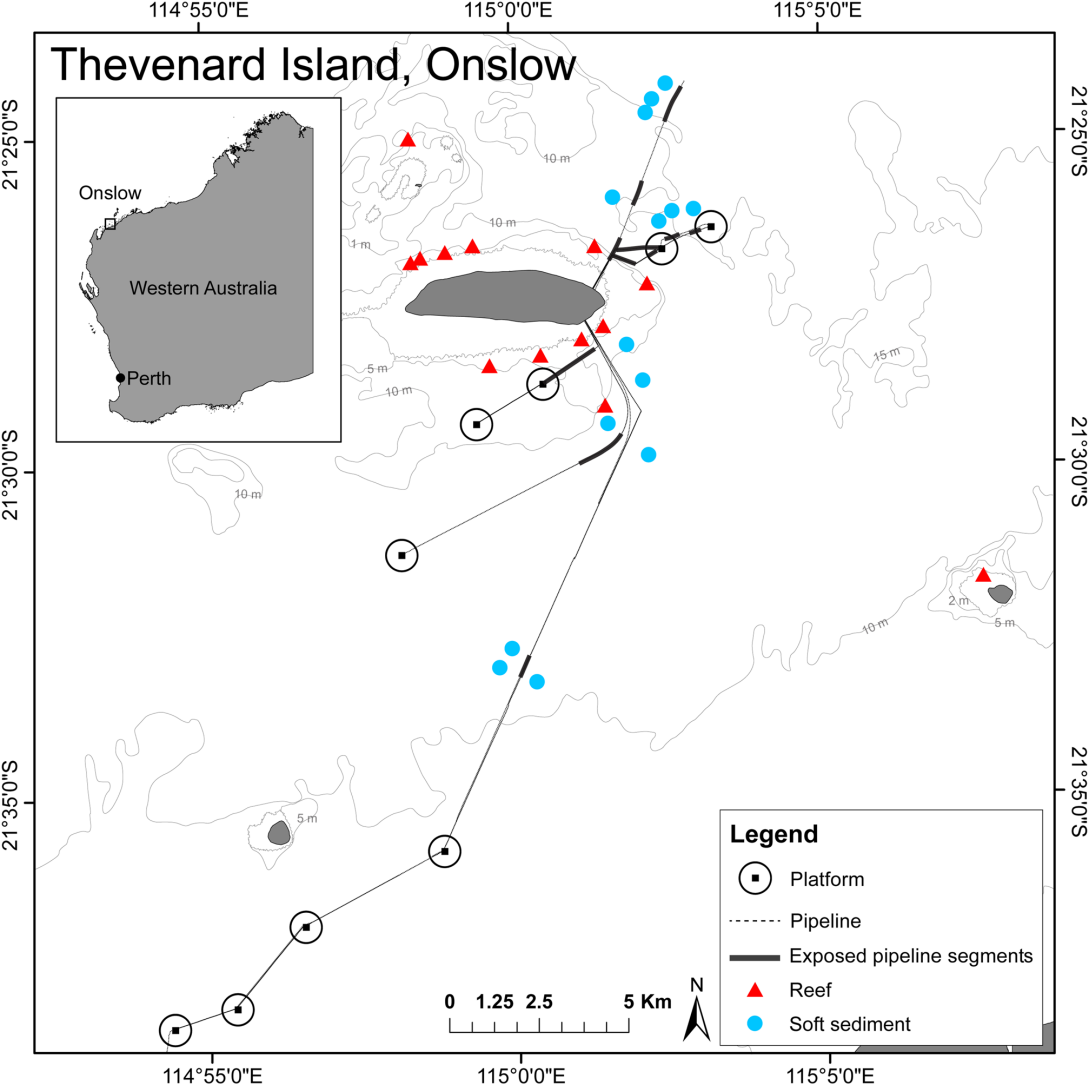
Location of exposed subsea pipelines surveyed and surrounding reef and soft sediment sites, in the vicinity of Thevenard Island, off Onslow, Western Australia (generated using ArcMap v10.7.1, https://desktop.arcgis.com, Memory-Map v1.2, https://memory-map.com, and Adobe Illustrator v25.0.1, https://www.adobe.com/au/products/illustrator.html).

**Figure 2:**
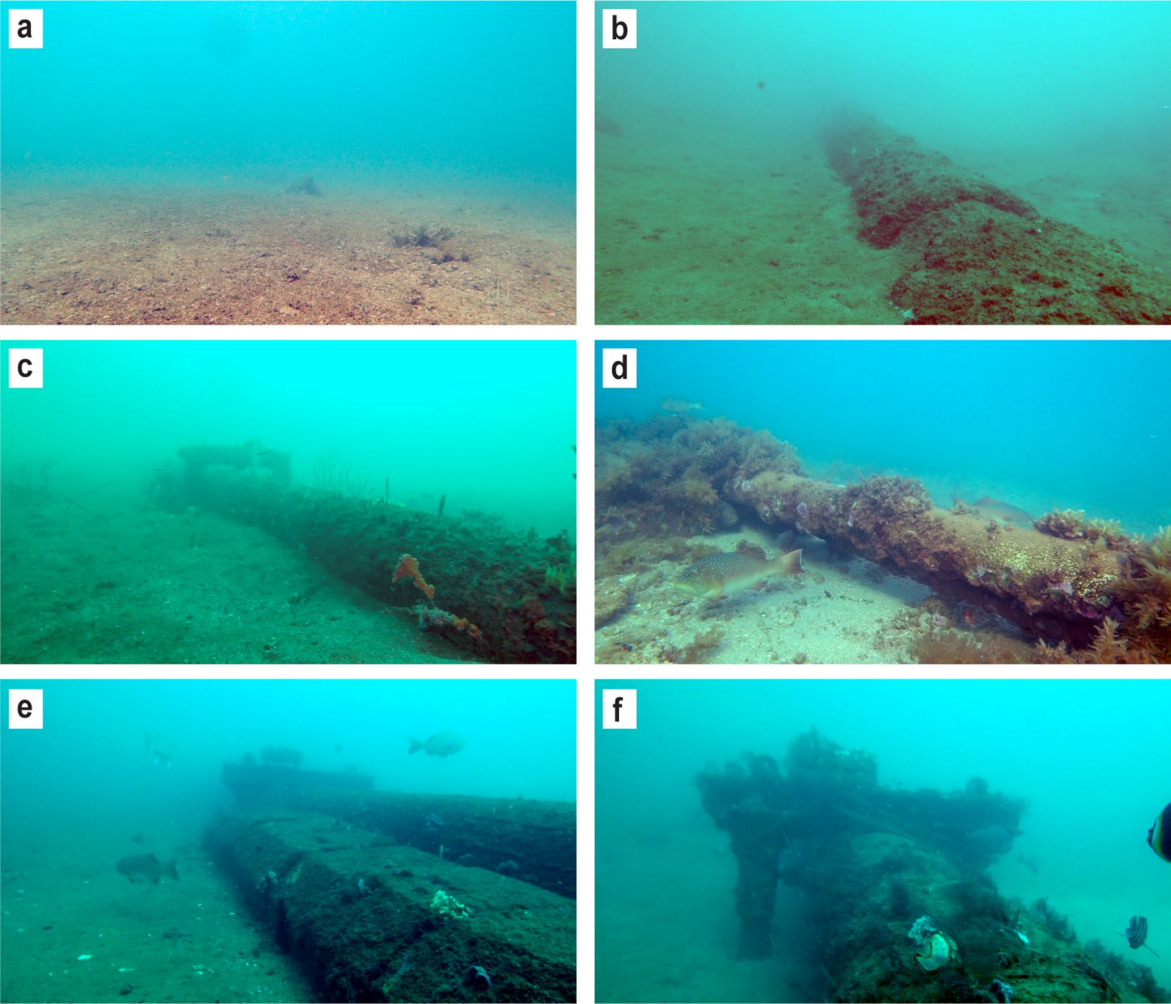
Representative (**a**) buried, (**b**) more than half buried, (**c**) more than half exposed, (**d**) spanning above the seafloor pipeline positions and associated structures along the pipe, (**e**) concrete mattress, (**f**) tie down (generated using Adobe Illustrator v25.0.1, https://www.adobe.com/au/products/illustrator.html).

Surveys were carried out in September 2018 on a network of subsea pipelines located near Thevenard Island, Western Australia, ranging in water depths of 10.6–20 m (Fig. 1). The majority of the pipelines were installed between 1989 and 1994, with the most recent installation in 2001 prior to cessation of operations in 2014. The network of pipelines has a combined length of 132 km in depths ranging from 0 to 20 m and connects nine platforms (three tripods and six monopods) to onshore facilities. During installation, ~ 80% of the pipelines were trenched and backfilled. At the time of the study approximately 14 km (~ 10%) of pipelines were exposed above the substrate and ranged from more than half-buried, more than half-exposed to spanning above the seafloor (Fig. 2b–d). The proportion of pipeline buried (Fig. 2a) and how exposed above the seabed unburied sections are is likely to change over time (especially in high current areas) due to sediment transport and scouring processes in the shallow-water environment. For the purposes of this study, other structures associated with the pipeline such as concrete mattresses and tie downs were considered part of the pipeline (Fig. 2e,f). Fish seen on these structures were included in counts from the pipelines. The outer diameter of the pipelines ranged between approximately 89–720 mm. Marine growth had not been cleaned from the pipelines since installation, but had been subject to natural disturbances such as cyclones.

**Figure 3:**
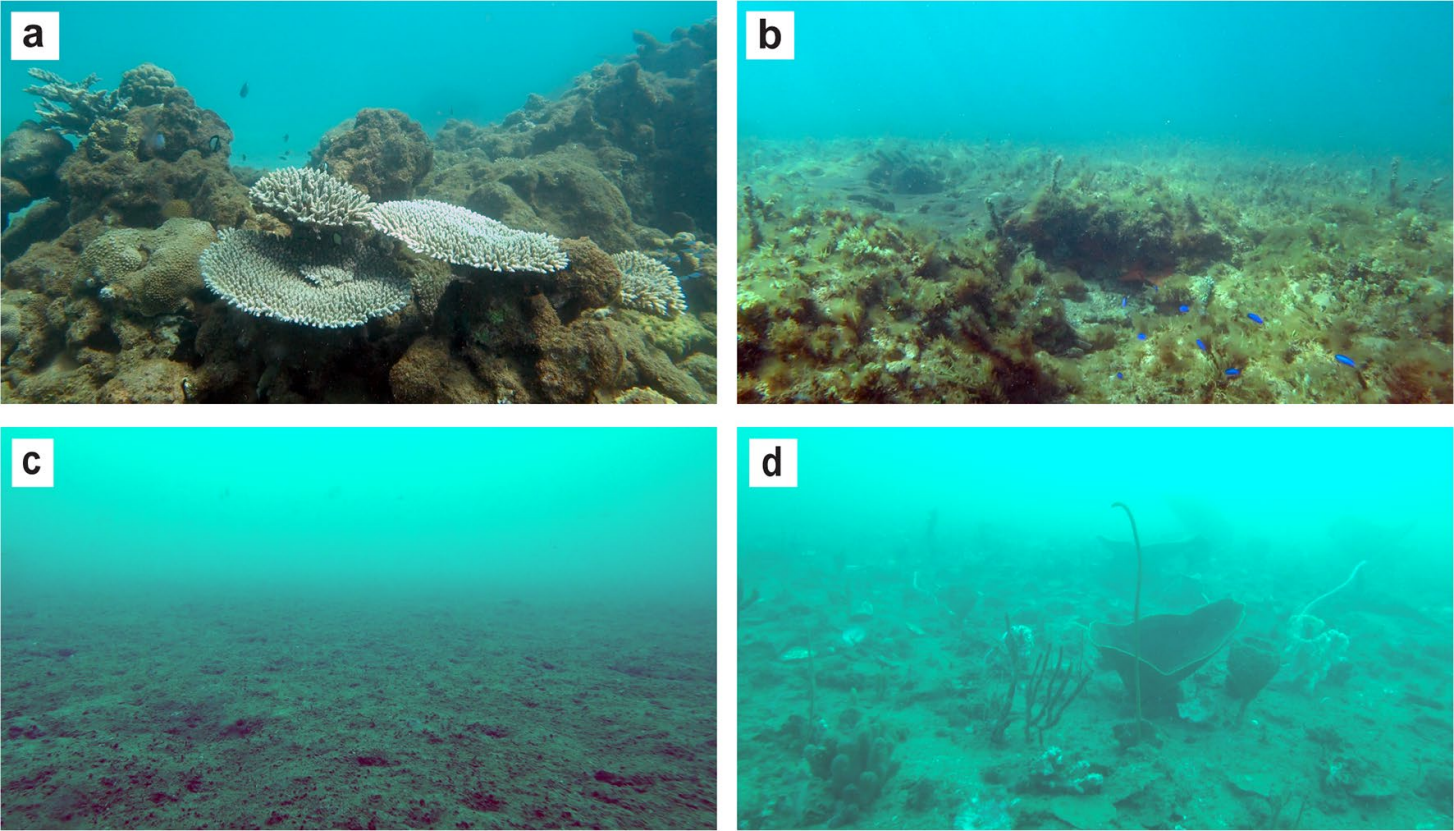
Representative reef (**a**,**b**) and soft sediment (**c**,**d**) habitats surrounding pipelines in the area around Thevenard Island, Western Australia (generated using Adobe Illustrator v25.0.1, https://www.adobe.com/au/products/illustrator.html).

We also surveyed natural reefs and soft sediment habitats in the surrounding area in water depths of 3.7–18.5 m at the same time to provide ecological context to the data obtained from the pipelines. GIS maps of the region with habitat overlays were used as guides for the selection of reef and soft sediment sites^41^. Reef habitat consisted of hard substrate with coral cover and/or macro algae (Fig. 3a,b), while soft sediment consisted of bare sand (Fig. 3c) or sand with patchy epibenthos (e.g. sponges and gorgonians) with underlying hard substrate not visible (Fig. 3d). The inshore waters where the focal pipelines are located are closed to commercial fisheries, except for a small-scale trawl fishery that targets banana prawns (*Fenneropenaeus indicus*) and a pelagic fishery for Spanish mackerel (*Scomberomorus* spp.)^42^. This region is subject to recreational fishing activities, however due to low human population sizes along the Pilbara coast, fishing pressures are minimal^43^.

### Sampling technique

Two mini ROVs of a similar size and functionality (SeaBotix vLBV300: 625 mm × 390 mm × 390 mm (l × w × h), ~ 18 kg; and BlueROV2 Heavy Configuration; 457 mm × 338 mm × 254 mm, ~ 11 kg) fitted with a stereo-video system^44,45^ were used to survey fish associated with pipelines, natural reefs and soft sediment habitats. The two ROVs were operated simultaneously, with the SeaBotix vLBV300 used to survey pipelines and the BlueROV2 used in natural habitats. The stereo-video systems on both ROVs used Sony FDR-X3000 ActionCams mounted in purpose-built housings with a base separation of 590 mm and an inward convergence of 5°. Cameras were set to record at 60 frames per second in a 1080p format. The SeaBotix vLBV300 ROV used a Tritech Ultra Short Baseline Positioning system (USBL) and an Oculus 750D multibeam sonar to help with positioning and navigation, while the BlueROV2 was equipped with Seatrac X150 USBL and X010 Modem.

### Pipeline surveys

GIS maps combined with recent hydrographic survey data were used to identify the position of exposed segments of pipeline around Thevenard Island. Live feed from the ROV camera and attached multibeam sonar were then used to locate pipelines *in-situ*. Once located, the ROV operated approximately 1.4 ± 0.05 m from the pipeline on one side only, with the system angled approximately 25° (23.05 ± 0.77°) towards the pipeline to enable a field of view of any undercut sections between the pipe and the seafloor. The system was operated at an average flight speed of approximately 0.54 ± 0.04 m/s (similar to the recommended velocity of stereo-DOV transects, 0.3 m/s^45^). Continuous footage of exposed pipelines was collected during active boating, with the vessel trailing behind the stereo-ROV, 100–150 m away. In total, eleven segments of exposed pipeline were surveyed with segment lengths varying between 0.3 and 1.7 km, which was dependent on the level of exposure of the pipeline. Pipeline surveys were completed between 08:30 and 17:00 h to minimise the effects of diel changes in fish behaviour on data collected^31,46^.

Quantitative comparisons between the reef and soft sediment habitats were undertaken by dividing continuous footage of the pipelines into 50 m transects, with a minimum 20 m separation between transects to ensure independence. To do this, the average flight speed of the stereo-ROV for each segment of pipeline was used to determine the time taken to complete a 50 m transect. Each pipeline transect surveyed encompassed an area 5 m wide × 50 m long (250 m^2^). The level of pipeline exposure varied across transects. If a 50 m transect contained more than 17.5 m (35% of a 50 m transect) of buried pipeline, it was excluded from image analysis. In total, 88 independent 50 m transects were retained for analyses. For pipeline segments that were included in analyses, on average per transect: 5% (2.33 ± 0.72 m) was in free-span above the seafloor, 55% (27.62 ± 2.10 m) was more than half-exposed, 33% (16.40 ± 2.02 m) was more than half-buried and 7% (3.66 ± 0.93 m) was completely buried.

### Reef and soft sediment surveys

Concurrent surveys in natural habitats were undertaken > 500 m away from the pipeline or any artificial structure, such as platforms. The ROV was operated from an anchored vessel and was continuously flown for approximately 25 min. As the vessel was stationary during these surveys, the operating range of the stereo-ROV was limited by the tether length (150 m). To avoid tension on the tether and ensure new ground was covered, the ROV was maneuvered in an expanding square around the vessel (similar to polygonal patterns described in Ref.^47,48^). During image analysis, the imagery was split into 5 m × 50 m transects (250 m^2^) with a 20 m separation between the end and start of transects as per the pipelines. Determining the start and end of these transects followed the procedure described above for pipeline transects. Imagery was analysed from 150 transects derived from 13 sites in reef habitat, and 145 transects from 14 sites in soft sediment habitat. Surveys in natural habitats were similarly undertaken between 08:30 and 17:00 h. All fish surveys were completed in compliance with the Australian Code for the Care and Use of Animals for Scientific Purposes and were approved by the Curtin University Animal Ethics Committee (ARE_2018_20).

### Stereo-video calibration and video analysis

Stereo-video systems were calibrated before and after fieldwork using the software package ‘CAL’ (https://www.seagis.com.au/bundle.html) following well established protocols and guidelines in Ref.^49–52^. All fish counts, identifications, and fork length (FL) measurements (tip of snout to mid of forked caudal fin) were made in EventMeasure Stereo Version 5.25 (https://www.seagis.com.au/event.html). Where fish could not be identified to a species level, individuals were pooled to the next highest taxonomic level, i.e. genera/family. To maintain a defined unit area of sampling across image analyses a horizontal and vertical constrained field of view was set to 2.5 m in either direction of the centre point (x = 5 m, y = 5 m), with a depth (z) range to 7 m. All individuals were counted within this defined sampling area, and those that were observed outside this area were not included in the data set^45^. Fish that were identified as having left the area of the transect, which later re-entered (where they could be identified) were only counted once. In order to obtain positioning and time information, the video imagery from the stereo system was synchronised with the video footage collected by the onboard ROV camera. Synchronisation was achieved by referring to the timecode overlay and manually defining the start time of the ROV footage. Both the ROV footage and stereo footage were then paused at the same unique synchronisation point (i.e. a digital clapperboard or physical clap). Calculating the difference in the elapsed time between the stereo and ROV video footage allowed us to define the start time of the high definition footage. Using the event logs collected in the field we were able to skip to the time at which the ROV commenced its survey. For quantitative analyses, this also allowed us to identify fish counts and measurements that were observed within the timed 50 m transects using their corresponding time stamp.

### Calculating biomass, feeding guilds and economic value of fish

Fish length was used as a proxy of weight (biomass), using the equation: Weight (g) = a × Length (cm)^b^^53^. Relevant slope (a) and intercept (b) values for different species/genera were sourced from FishBase^54^. Where fish could not be measured due to visual obstruction from other fish or structure (pipe and reef etc.), or were oblique to the camera with neither the head nor tail visible, we allocated that fish a mean length which was based on that specific species from within the same habitat (similar to Ref.^55^). Classification of feeding guilds for fish were sourced from FishBase based on the ecology and/or diet descriptions^54^ (See Supplementary Table S1). The value of commercial and recreational fish ($AUD/kg) was calculated using the mean wet weight market value for commercial species for 2017/2018^42^. In total, 39 species for which a corresponding market value was available were recorded (See Supplementary Table S2).

### Statistical analysis

Statistical analyses were undertaken in PRIMER 7 with PERMANOVA + add on^56^. A one way PERMANOVA was used to test for differences among habitats in the numbers of species, overall fish abundance, the overall biomass, differences in feeding guilds and focal species (*Habitat*: 3 levels; pipeline, reef, soft sediment)^56,57^. Focal species were considered to be those species identified as being dominant in a SIMPER analyses, as well as those species commonly targeted by fishers in the Pilbara area^43^. Focal species were *Neopomacentrus aktites* (Western Australian demoiselle), *Pomacentrus coelestis* (Neon damsel), *Thalassoma lunare* (Moon wrasse), *Parupeneus indicus* (Yellowspot goatfish), *Scarus ghobban* (Bluebarred parrotfish), *Pentapodus porosus* (Northwest threadfin bream), *Choerodon cauteroma* (Bluespotted tuskfish)*, Choerodon schoenleinii* (Blackspot tuskfish), *Plectropomus* spp. (Coral trout), *Lutjanus carponotatus* (Stripey snapper) and *Lethrinus laticaudis* (Grass emperor). Data were tested for dispersion using PERMDISP^58^ and were analysed using the untransformed data based on a Euclidean distance matrix. When a statistical difference was found (*P* < 0.05, using 9999 permutations), a post-hoc pairwise comparison was completed. *P* values from pairwise tests are indicated using P_(pairwise)_. A Monte Carlo bootstrapping correction was used in instances where a low permutation value was obtained for post-hoc tests (< 100), and indicated using P(MC). A venn diagram showing the number of species shared between habitats was constructed.

To test for statistical differences in the fish assemblage recorded between habitats a one-way PERMANOVA was used (*Habitat*: 3 levels; pipeline, reef, soft sediment). A fourth root transformation was applied to down weight the influence of more common species over those rarely recorded across the data set. A Bray Curtis similarity matrix was used for analysis with a dummy added variable (+ 1) to account for transects in which no fish were observed. A significant difference was determined when *P* < 0.05 using 9999 permutations, followed by a pairwise comparison to distinguish which habitats were statistically different from one another (P_(pairwise)_). Principal coordinates analysis (PCO) and constrained canonical analysis of rincipal coordinates (CAP) plots were used to visually represent differences in the abundance and biomass of fish assemblages among habitats. Overlays onto CAP axes were done using SIMPER analyses and selecting the top five species that contributed to group differences (based on similarity/standard deviation values). A leave-one-out allocation test was also used to estimate and classify how distinct samples were relative to each habitat.

## Results

**Figure 4:**
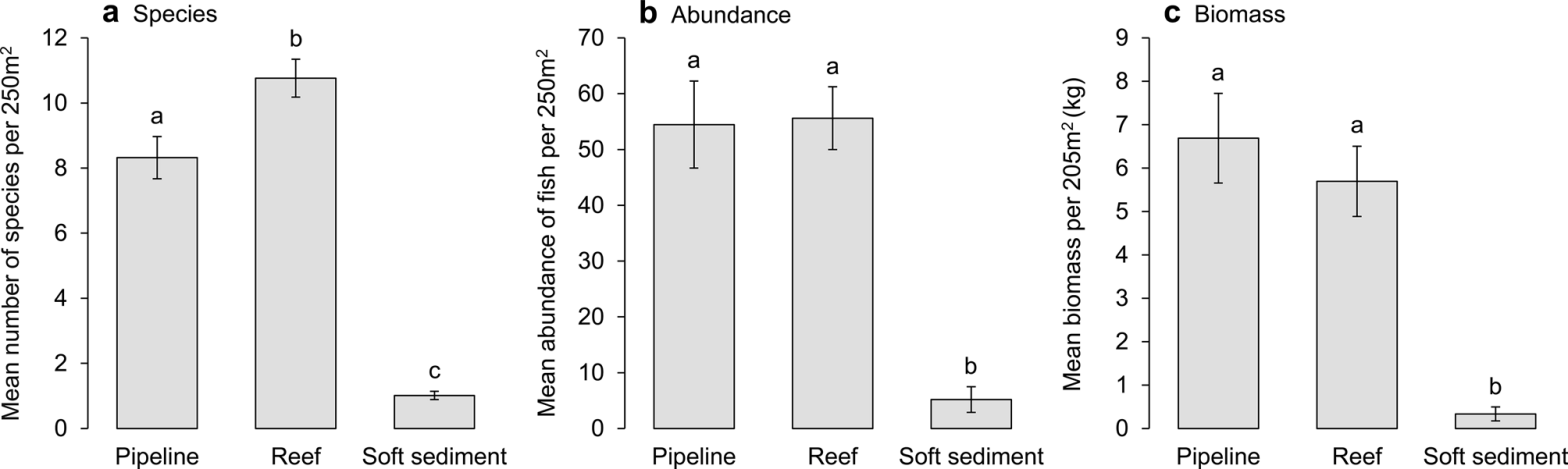
Mean (± SE) number of species (**a**), abundance (**b**), and biomass of fish (kg) (**c**) per transect (50 m × 5 m, 250 m^2^) for pipeline, reef, and soft sediment habitats. Statistically similar means are indicated by the same letter (e.g. a) (generated using Microsoft Excel v16.0.5122.1000, https://www.microsoft.com/).

**Figure 5:**
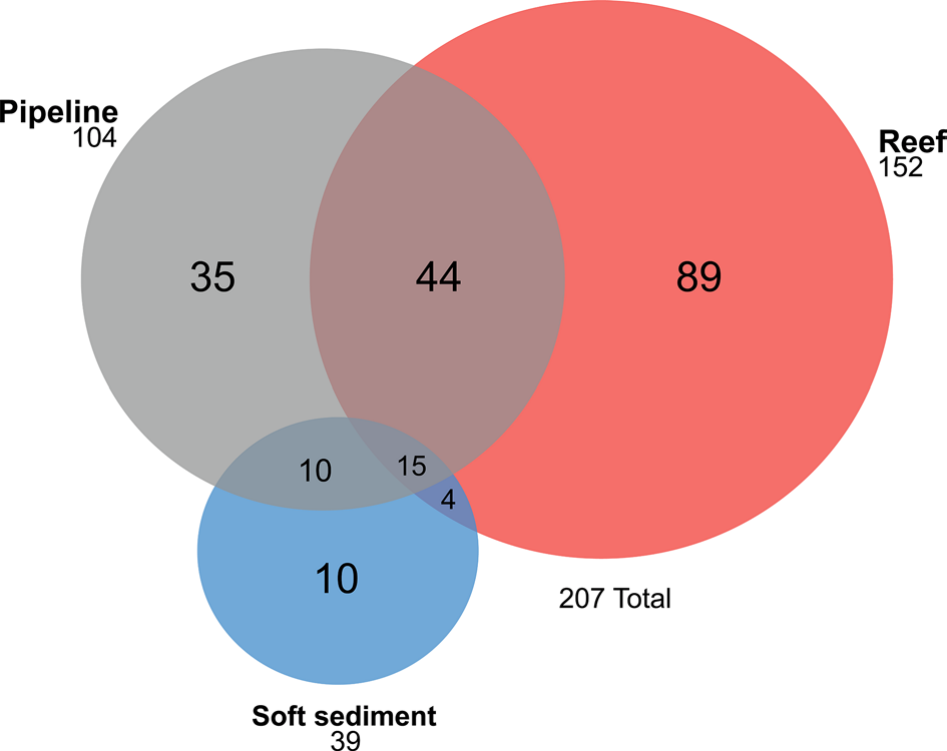
Total number of species recorded at each habitat: pipeline, reef, soft sediment, and across combinations of habitats (generated using Venn Diagram Plotter v1.5.5228.29250, http://omics.pnl.gov/).

A total of 13,883 fish from 46 families and 207 species were recorded in surveys of pipelines and surrounding reef and soft sediment habitats. The mean number of species varied between habitats (PERMANOVA: F_2,382_ = 125.82, *P* < 0.001) with reef having significantly more species than pipeline (P_(pairwise)_ = 0.008) and soft sediment habitats (P_(pairwise)_ < 0.001), which were also different from one another (pipeline and soft sediment: P_(pairwise)_ < 0.001) (Fig. 4a). Pipeline and reef habitats however were more similar in the composition of the fish assemblage, sharing 44 species (21% of the total fish assemblage), than pipeline and soft sediment habitats, which shared only ten species (5%) (Fig. 5). Fish abundance varied between habitats (PERMANOVA: F_2,382_ = 33.339, *P* < 0.001), but pipeline and reef habitats had similar abundances of fish (P_(pairwise)_ = 0.904). Soft sediment habitat had lower abundances of fish than pipeline or reef habitats (P_(pairwise)_ < 0.001) (Fig. 4b). A similar pattern was observed for biomass (PERMANOVA: F_2,382_ = 24.641, *P* < 0.001), with soft sediment having lower biomass compared to pipeline and reef habitats (P_(pairwise)_ < 0.001), which were similar (P_(pairwise)_ = 0.461) (Fig. 4c).

### Fish assemblage composition

**Figure 6:**
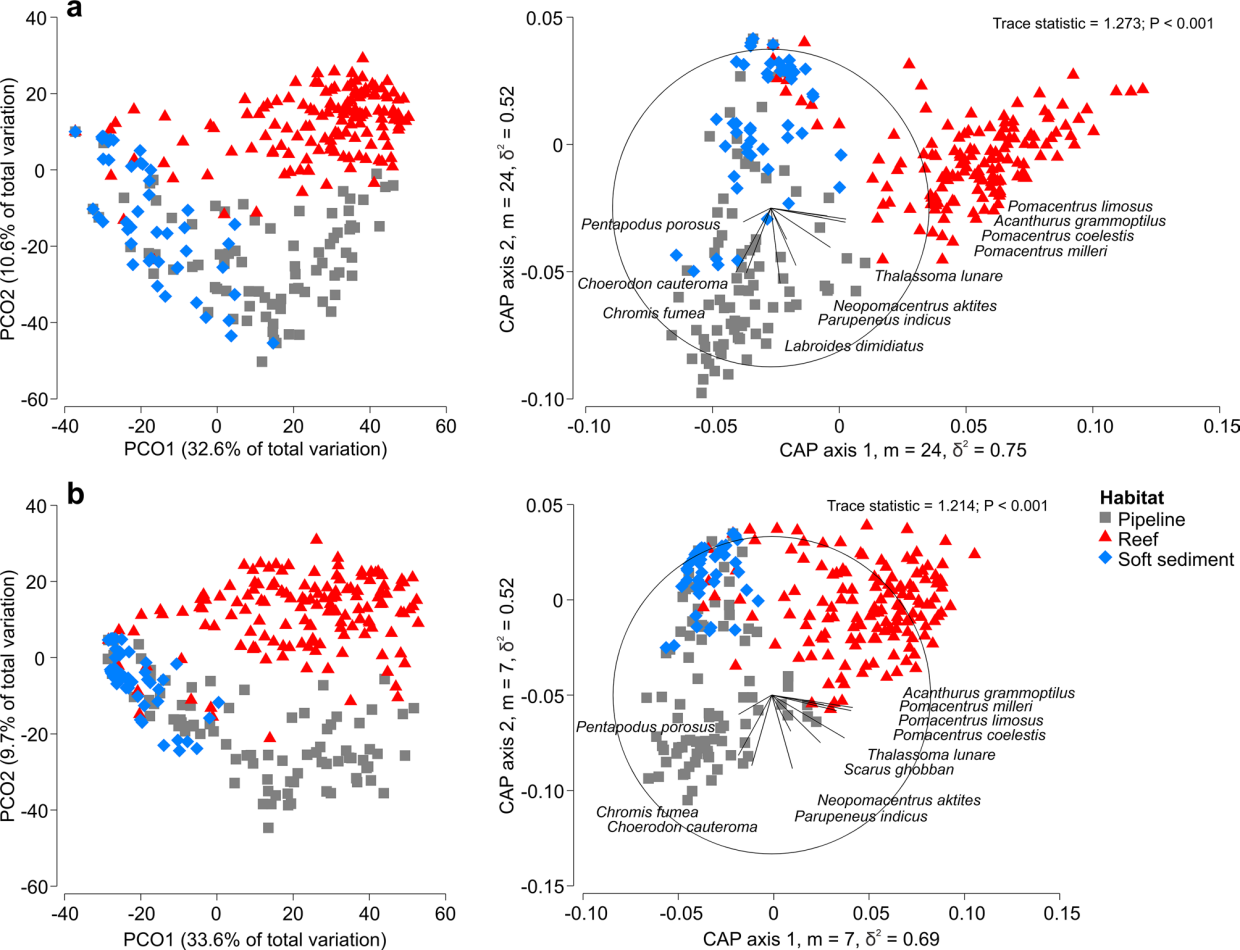
Principal Coordinates Analysis (PCO) and Canonical Analysis of Principal Coordinates (CAP) plots for the abundance (**a**) and biomass (**b**) of the observed fish assemblage with SIMPER species overlay showing the differences between habitats: pipeline, reef, and soft sediment. Ordinations are based on four root transformations and Bray Curtis similarities (generated using PRIMER 7 v7, https://www.primer-e.com/).

The composition of the fish assemblage differed among habitats (PERMANOVA: F_2,382_ = 64.833, *P* < 0.001) with each habitat being distinct from one another (P_(pairwise)_ < 0.001). The leave one out allocation success between pipeline, reef, and soft sediment habitats was high overall with 85.38% of samples (327/383) being correctly classified to the correct habitat (Table 1). However, there was higher overlap between pipeline and soft sediment habitats, than between reef and pipeline habitats (Table 1). A PCO and CAP plot showed a distinct separation between reef and the other habitats (pipeline and soft sediment), which were more similar, but still statistically different from one another (*P* < 0.001; Fig. 6a). This separation was driven by high abundances of damselfish species (*Pomacentrus limosus, P. coelestis,* and *Pomacentrus milleri*), *Acanthurus grammoptilus* and *T. lunare* that were observed on natural reefs. Separations between pipeline and soft sediment were less distinct on CAP axis 2 and were driven by fish that are reef associated (*Chromis fumea, N. aktites,* and *Labroides dimidiatus*). The separation was also driven by fish that occupy a combination of sandy areas over or near reef areas (*C. cauteroma*, *P. indicus,* and *P. porou*s) (Fig. 6a). A similar pattern was detected for biomass at an assemblage level (PERMANOVA: F_2,382_ = 63.303, *P* < 0.001), where all habitats differed from one another (P_(pairwise)_ < 0.001) with an overall high (86.16%) allocation success for samples by habitat (Table 1). Separation and grouping by habitat in the ordinations was driven by high abundances of *P. porosus, C. cauteroma and C. fumea* on the pipeline, and larger *P. indicus* individuals, which created a greater biomass on pipelines than in natural reef and soft sediment habitats (Fig. 6b). Conversely, reef samples were driven by the high abundance of damselfishes (*P. milleri*, *P. limosus* and *P. coelestis*), as well as *A. grammoptilus,* and *T. lunare*, which were more abundant on the reef in comparison to pipeline and soft sediment habitats (Fig. 6b).

**Table 1:** Leave-one-allocation success of observations to habitat: cross validation for fish abundance and biomass.

Habitat	Abundance (m: 24, total correct: 327/383)					Biomass (m: 7, total correct: 330/383)				
	Pipeline	Reef	Soft sediment	Total	Success (%)	Pipeline	Reef	Soft sediment	Total	Success (%)
Pipeline	67	0	21	88	76.14	67	0	21	88	76.14
Reef	2	130	18	150	86.67	4	125	21	150	83.33
Soft sediment	14	1	130	145	89.66	7	0	138	145	95.17

### Feeding guilds

**Figure 7:**
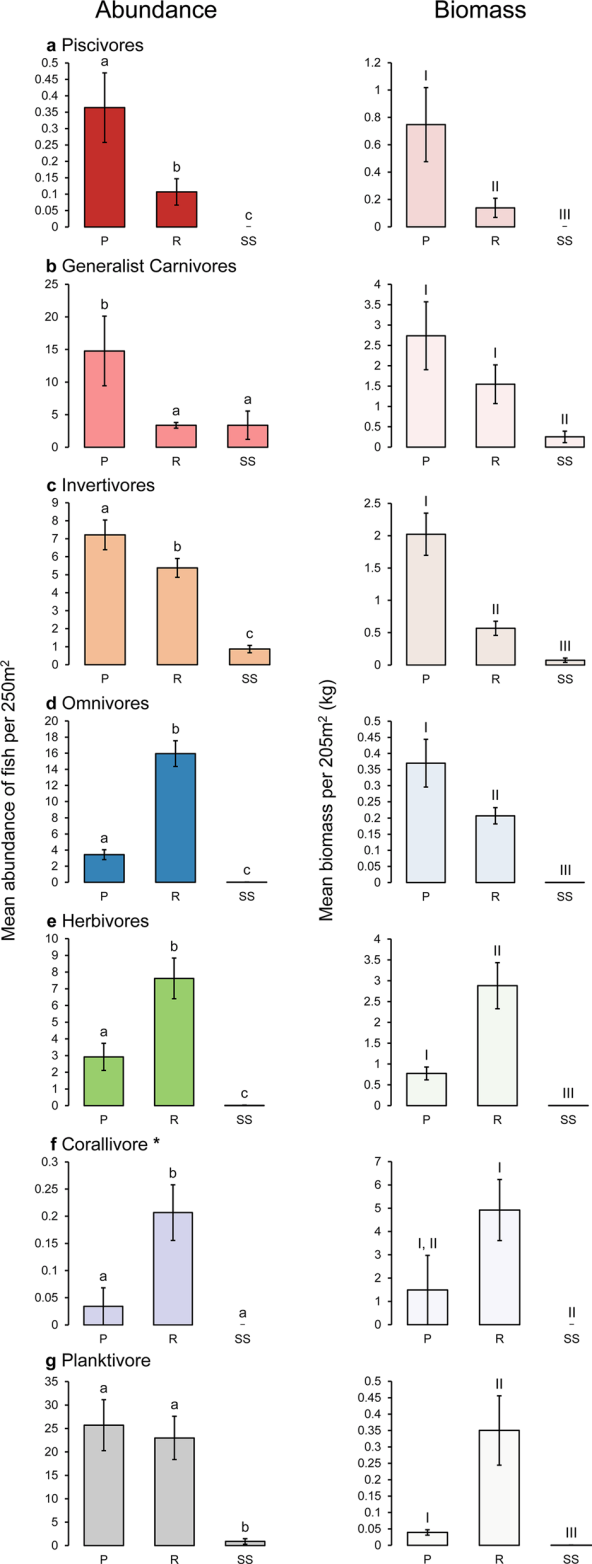
Mean (± SE) abundance and biomass of fish per transects (50 m × 5 m × 5 m) for feeding guilds: piscivores (**a**), generalist carnivores (**b**), invertivores (**c**), omnivores (**d**), herbivores (**e**), corallivores (**f**), and planktivores (**g**), between habitats: pipeline (P), reef (R), soft sediment (SS). Statistically similar means are indicated by the same letter for abundance (e.g. a), and roman numerals for biomass (e.g. I). *Biomass of corallivores are represented in grams (g) (generated using Microsoft Excel v16.0.5122.1000, https://www.microsoft.com/).

Guild-specific abundance was greater on pipelines than in reef habitats for piscivores (P(MC)_(pairwise)_ = 0.009; Fig. 7a), generalist carnivores (P_(pairwise)_ < 0.001; Fig. 7b), and invertivores (P_(pairwise)_ = 0.048; Fig. 7c), while similar abundances were found between these habitats for planktivores (P_(pairwise)_ = 0.727; Fig. 7g). Biomass was greater on pipelines than in reef habitats for piscivores (P_(pairwise)_ = 0.006; Fig. 7a) and invertivores (P_(pairwise)_ < 0.001; Fig. 7c), but similar for generalist carnivores (P_(pairwise)_ = 0.195; Fig. 7b) and corallivores (P_(pairwise)_ = 0.092; Fig. 7f). Reef habitats had greater abundances of omnivore (P_(pairwise)_ < 0.001; Fig. 7d), herbivores (P_(pairwise)_ = 0.002; Fig. 7e) and corallivores (P(MC)_(pairwise)_ = 0.016; Fig. 7f) than pipeline habitats. A greater biomass of herbivores (P_(pairwise)_ = 0.001; Fig. 7e) and planktivores (P_(pairwise)_ = 0.010; Fig. 7g) was found in reef habitats compare to pipeline habitats. This differed for omnivores where a greater biomass was found on pipeline habitats (P_(pairwise)_ = 0.014; Fig. 7d). In general, abundance and biomass of all guilds were lowest in soft sediment habitats (P_(pairwise)_ < 0.05), with the expectation of the abundance of generalist carnivore, which was similar to reef (P_(pairwise)_ = 0.999; Fig. 7b), and the abundance and biomass of corallivores, which were similar to pipeline (P(MC)_(pairwise)_ = 0.195; P(MC)_(pairwise)_ = 0.202; Fig. 7f).

### Focal species

**Figure 8:**
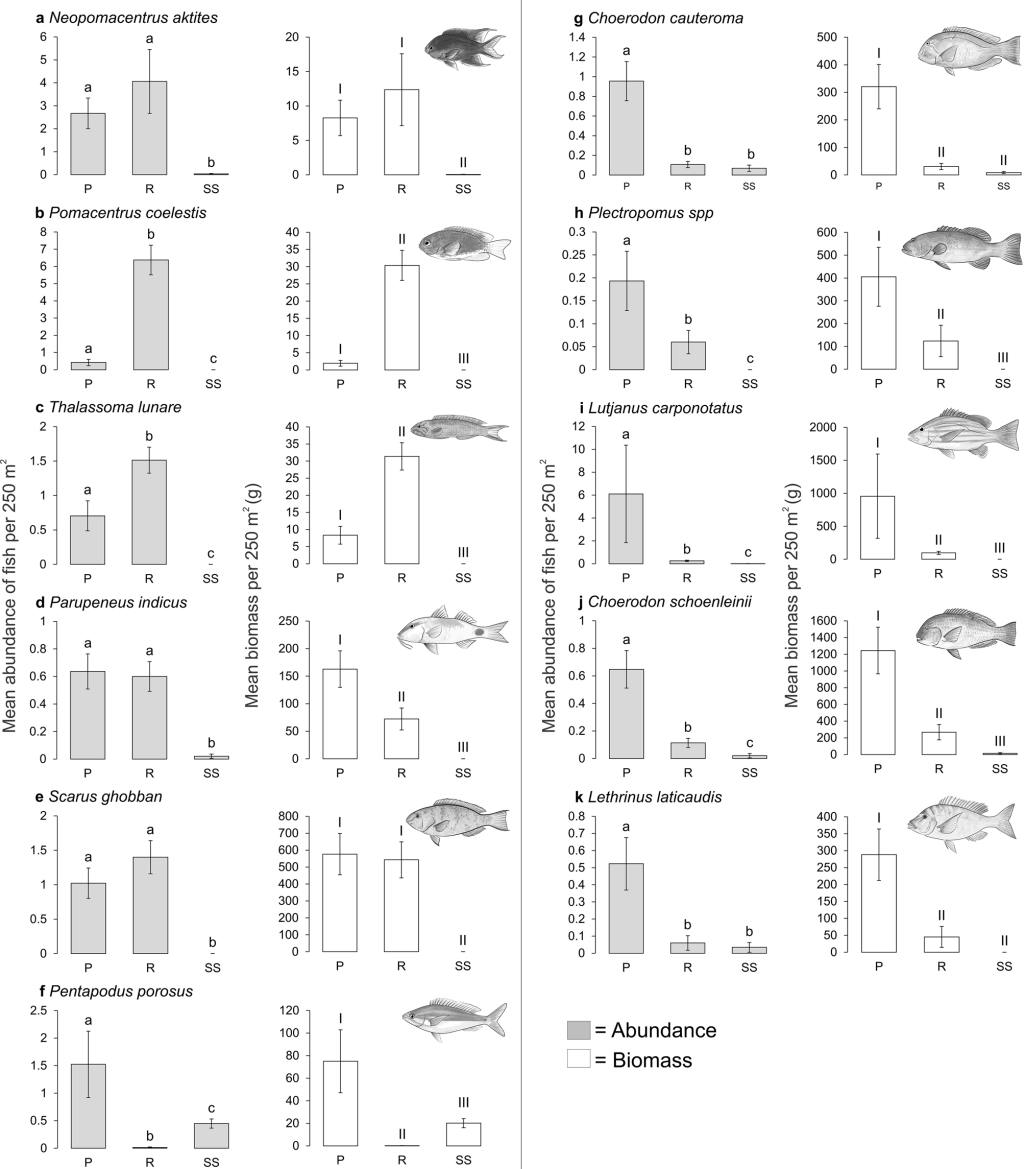
Mean (± SE) abundance and biomass of *Neopomacentrus aktites* (**a**), *Pomacentrus coelestis* (**b**), *Thalassoma lunare* (**c**), *Parupeneus indicus* (**d**), *Scarus ghobban* (**e**), *Pentapodus porosus* (**f**) *Choerodon cauteroma* (**g**), *Plectropomus* spp. (**h**), *Lutjanus carponotatus* (**i**), *Choerodon schoenleinii* (**j**), *Lethrinus laticaudis* (**k**) among habitats: pipeline (P), reef (R), soft sediment (SS). Statistically similar means are indicated by the same letter for abundance (e.g. a), and roman numerals for biomass (e.g. I) (generated using Microsoft Excel v16.0.5122.1000, https://www.microsoft.com/).

The mean abundance and biomass of focal species differed between habitats (PERMANOVA: *P* < 0.05; Fig. 8). For *N. aktites*, *P. indicus* and *S. ghobban* the mean abundance was similar between pipeline and reef habitats (P_(pairwise)_ = 0.565; P(MC) _(pairwise)_ = 0.835; P_(pairwise)_ = 0.309, respectively) with both habitats having a greater abundance of fish than soft sediment habitat (P_(pairwise)_ < 0.001; Fig. 8a,d,e). A similar pattern was observed for the biomass of these species, with the exception of *P. indicus* where a greater biomass was recorded on the pipeline in comparison to reef (P_(pairwise)_ = 0.010) and soft sediment (P_(pairwise)_ < 0.001), which also differed from one another (P_(pairwise)_ < 0.001; Fig. 8d). By contrast, the mean abundance and biomass of *P. coelestis* was greater in reef habitat than on the pipeline (P_(pairwise)_ < 0.001), and soft sediment habitats (P_(pairwise)_ < 0.001) where no individuals were encountered (Fig. 8b). A greater abundance and biomass of *T. lunare* was detected on reefs than pipelines (*P* < 0.05) and soft sediment habitats (P_(pairwise)_ < 0.001), which also differed from one another (P_(pairwise)_ < 0.001; Fig. 8c). A greater abundance of *P. porosus* was found on the pipeline in comparison to natural habitats (reef: P_(pairwise)_ > 0.001; soft sediment: P_(pairwise)_ = 0.002), where soft sediment had a higher abundance than reef habitat (P(MC)_(pairwise)_ < 0.001; Fig. 8f). A similar pattern was observed for the biomass of *P. porosus* (Fig. 8f)*.*

Species that are commonly targeted or retained by recreational fishers in the Pilbara region (*C. cauteroma*, *C. schoenleinii*, *Plectropomus* spp., *L. carponotatus*, and *L. laticaudus*) were more abundant on pipelines than reef and soft sediment habitats (P_(pairwise)_ < 0.05; Fig. 8g–k), which were also different from one another. *C. cauteroma* and *L. laticaudus* were exceptions where reef and soft sediment had a similar abundance of individuals (P(MC) _(pairwise)_ = 0.414; P(MC) _(pairwise)_ = 0.623, respectively; Fig. 8g,k), which were less than the pipeline. The biomass of these species was also consistently greater on pipelines than in natural habitats (P_(pairwise)_ < 0.05; Fig. 8g–k) with soft sediment having a lower biomass than reefs. Again, *C. cauteroma* and *L. laticaudus* were exceptions with natural habitats having a similar biomass of fish (P_(pairwise)_ = 0.056; P(MC)_(pairwise)_ = 0.153, respectively; Fig. 8g,k) which were less than the pipeline.

### Economic value

An equivalent area (250 m^2^) of pipeline had ~ 71% more biomass (3.5 times more) than natural reefs, and ~ 98% more biomass (44.5 times more) than soft sediment habitat for species commonly retained by commercial and recreational fishers in the region (See Supplementary Table S2). When converted into an economic dollar value ($AUD) based on market prices for wet weight (See Supplementary Table S2), an equivalent area of pipeline contained an economic value of fish that was 3.4 times greater than the adjacent natural reef habitat and 57 times more than the adjacent soft sediment habitat (Table 2).

**Table 2:** Economic value of species retained by commercial and recreational fishers.

	Pipeline	Reef	Soft sediment
Mean biomass per 250 m^2^ (kg)	4.90 ± 0.92	1.40 ± 0.25	0.11 ± 0.07
Mean catch value per 250 m^2^ ($AUD)	30.20 ± 4.76	8.82 ± 1.88	0.53 ± 0.31
Number of transects	88	150	145

## Discussion

Fish assemblages associated with pipelines were distinctly different from nearby natural reefs and soft sediment habitats. Pipelines were characterised by a greater abundance and/or biomass of species from higher trophic levels (i.e. piscivores, generalist carnivores, and invertivores) compared to reef and soft sediment habitats, which resulted in them having a higher fisheries value than equivalent areas of reef and soft sediment. In comparison, natural reefs had a greater proportion of omnivores, herbivores, and corallivores than pipelines and soft sediment habitats, which was likely due to the greater cover of benthic communities and associated food sources (i.e. coral and macroalgae) observed on reefs. Soft sediment habitat was dominated by generalist carnivores and invertivores, but overall had much lower abundances than other habitats.

These findings differ from a previous study undertaken in the same region at similar depths, which indicated that fish assemblages were similar in both the pipeline and soft sediment habitats^30^. However, the disparity in findings between this study and that of Bond et al.^30^ are likely attributable to the sampling technique (stereo-BRUVs) attracting fish from other habitats. For example, attracting fish from the soft sediment habitat to the baited camera located near the pipeline. Stereo-BRUVs have been shown to sample a broad range of species^59,60^, and are particularly effective at sampling large, highly mobile carnivorous fishes. The distance these species travel to a bait is unknown^12^, and fish do tend to aggregate around the bait in numbers which are much higher than count data collected by other sampling techniques^60,61^. By comparison, the stereo-ROV collects data at a much finer scale than stereo-BRUVs and is particularly good for habitat affiliated fishes^12^. It is acknowledged that the stereo-ROV technique may have some avoidance biases due to the noise associated with the ROV thrusters, electronics and tether vibrations^12^. This avoidance may have been heightened within soft sediment habitat where there was limited structure for fish to take shelter. Consequently, it is possible that in soft sediment habitat fish avoided the ROV, increasing the difference between soft sediment areas and pipelines^12^.

Density-dependent mechanisms, such as habitat availability, competition, and predation have likely contributed to the observed abundance of fish along the pipeline. The presence of predatory fish on artificial reefs has been associated with food availability^24^, both on and off the structure, where searching for prey is likely optimised with lower energy expenditure^62,63^. For resident predatory species, such as *Plectropomus* spp., the limited spatial area of the pipeline may enhance prey encounters, whereby food sources are potentially concentrated along the structure. This may also be true for invertivores that consume sessile invertebrates that were associated with pipelines and will likely be influenced by patterns in epifaunal growth^32^. Habitat forming biota, particularly sponges, support a range of marine fauna (e.g. fish, crustaceans and echinoderms) and likely contribute a link between species of a higher trophic level^64^.

Fish that use reefs for shelter by day, but forage in different habitats by night, such as in seagrass or macroalgae beds or open sand, are also likely to benefit from the physical presence of structure within open and sparse habitats, where suitable food resources may be prevalent^65^ as opposed to shelter sites in natural reef habitat which may be distant from foraging habitat. Networks of pipelines are typically situated on sandy substrates and foraging efficiency may be increased for some species that feed on infaunal burrowing organisms (e.g. crustaceans, polychaetes and molluscs). Lutjanid species display this foraging behaviour at night by migrating to nearby habitats away from reefs to feed on invertebrates^38,66–68^. Some lethrinid species also migrate away from reefs to forage over soft substrate during the night^39,67,69,70^. Similar diel variations have been documented on subsea pipelines, with fewer encounters of fish and number of species at night, compared to during the day using industry ROV footage^31,32^. Therefore, the high abundance of lujtanids and certain lethrinids on the pipeline may not be due to prey availability on pipelines, but rather due to the physical structure acting as a daytime shelter. Fish that forage in other habitats and return to pipelines for shelter may play an important role in concentrating nutrients around pipelines via waste excretion^31,71–73^, which may, in itself, result in increased species, abundance and biomass of a range of species. Foraging fish returning to the pipeline may also be preyed upon by resident piscivores, which in turn excrete nutrients at the pipeline. Future work assessing diurnal variations both on and off the pipeline will provide better insights into the behaviour of fish that occupy these structures during the day and their role in facilitating nutrient and energy transfer onto the pipeline from surrounding areas.

Prey availability, structural complexity, and habitat characteristics influence the distribution of reef fishes^74,75^. Pipeline features such as span length^32,34^, wellheads^76,77^ and field joints^33^ have been associated with high abundances of fish, particularly of predatory species (e.g. lujtanids, epinephelids, and sebastids). Our findings were consistent with previous subsea pipeline studies^29–34^, whereby predatory fish (e.g. in this study, *L. carponotatus* and *Plectropomus* spp.) were commonly observed near pipe spans or utilising small interstitial spaces created between concrete mattresses and the pipe. Ambush predators, such as *Plectropomus* spp., likely rely on structural features that limit visibility to prey, thus increasing capture success^78,79^. Juvenile *Plectropomus* spp. display a strong association with *Acropora* corals over sandy substrates as the morphological complexity of *Acropora* skeletons provides shelter^79^, and importantly this *Acropora* edge habitat has a variety of food sources, with a high prevalence of small cryptic fishes around the coral, and invertebrates in the sandy substratum^74,79^. Shelter size requirements are also likely to change as fish grow due to the effectiveness of shelter and ease of rapid escape from predators^80^. Although pipeline features, such as spans and concrete mattresses, may not represent typical refuges (e.g. live coral) for some predatory fish, their structural complexity may serve as favourable habitats for both access to prey and refuge from predators.

Population estimates of some commercially and recreationally important fish species are influenced by behavioural biases towards divers, particularly in areas exposed to fishing pressure where fish exhibit more pronounced avoidance responses^81–84^. Behavioural reactions of avoidance or attraction to ROVs vary and are likely to be species-specific^85^. It is possible that fish responses to the ROV varied across habitat types. In particular, natural reefs have high structural complexity, which allows fish to shelter and potentially be obscured in surveys. Likewise, flight responses as the ROV approaches may be heightened in open and sandy habitats due to the lack of structure^12^. As pipelines are often the only shelter in an open and sandy environment, any avoidance towards the ROV is likely to result in fish fleeing near the structure in close proximity, where they can still be observed in the video imagery^12^. However, this is likely to be dependent on whether cautious fish take shelter on the side of the pipeline where ROV operations are taking place, still permitting observations. In some cases, fish may flee to the opposite side of the pipeline and be out of the field of view, limiting pipeline fish counts to a more conservative estimate. This is likely to be more of an issue where segments of pipeline are fully exposed, creating a larger obstruction in video imagery, in comparison to free-spanning segments of pipeline where fish may still be captured in the field of view underneath the pipe. Therefore, predatory fish that are commonly targeted by fishers may have been underestimated in the reef and soft sediment habitats due to potential avoidance behaviours in areas of high structural complexity or lack of structure, but may also have been underestimated where pipelines were fully exposed and when fish fled to the opposite side of the structure out of view of ROV cameras.

Previous studies have documented that pipelines can provide habitat for a greater abundance of fish than adjacent soft sediment habitat^29,30^. However, to our knowledge, the present study is the first to demonstrate that pipelines can hold a similar abundance and biomass of fish per unit area compared to natural reefs. Furthermore, the biomass of species commonly targeted by fishers that were recorded on pipelines was approximately 3.5 times greater than natural reefs, highlighting the potential fisheries value of these structures^29–34,77^. Studies by Bond et al.^29,30^ reported a higher biomass of targeted fish species associated with pipelines compared to adjacent natural habitats (predominantly soft bottom areas), ranging from two to eight times greater. The present study demonstrated even greater disparities in biomass between pipeline and natural habitats (3.5 times more than reef and 44.5 times more than soft sediment) than previous studies. We also note that our pipeline estimates may be conservative as we effectively only surveyed one side of the larger pipelines. However, it is possible that fish estimates in reef habitat were also similarly conservative and potentially under represented due to fish seeking shelter in or around the reef, obscuring their view from the stereo-ROV^12^. These disparities are likely attributable to the biases of the sampling method (stereo-BRUVs) used by Ref.^29,30^ in comparison to the biases of the stereo-ROV method used in the present study (discussed in Ref.^12^).

Predatory fish, such as *L. carponotatus*, *L. laticaudus, C. schoenleinii, Plectropomus maculatus* and *Plectropomus leopardus,* are some of the most commonly retained species by recreational anglers in the Pilbara region^43^. The prevalence of these species associated with pipelines, which were higher in abundance and biomass than surrounding natural habitats, suggests that these structures offer an extractive value for fishers, similar to purposely deployed artificial reefs^86,87^. In the North Sea, oil and gas pipelines are commonly targeted by commercial fishers, with ~ 36% of trips taking place within 200 m of a pipeline, and > 1% which actively target these structures^88^. The predatory fish assemblages that characterise pipeline infrastructure in the Pilbara region of north-western Australia exhibit high ecological and socioeconomic value, indicating that retaining these structures in situ offers significant ecological and community benefits.

Whether the higher fish numbers and biomass we recorded on these oil and gas pipelines are caused by fish production or is due to attraction from other nearby habitats is not clear (the production vs. attraction debate^24^). This is partly due to difficulties involved in demonstrating an overall increase in regional fish biomass after the installation of such structures, whilst controlling for natural variation, external fishing pressures and possible immigration^7^. Claisse et al.^6^ demonstrated that the secondary production on oil and gas platforms in California was 27.4 times more than natural rocky reefs at similar depths. To assess secondary production (the formation of new animal biomass from growth for all individuals in a given area during a period of time) we would need to resample these pipelines repeatedly. However, given these structures have been in situ for 17–29 years (at the time of this study), it is likely that they contribute directly to biomass production rather than simply attraction from the surrounding area. Removal of these structures would therefore likely result in a net habitat loss, resulting in a net loss of production in this region.

Identifying particular habitat features of pipelines that drive fish associations would be of benefit for decommissioning of structures, including planning and understanding their ecological value. The distinct fish assemblage observed on the pipelines suggests these structures are not surrogates of natural reefs or soft sediment habitats, but may offer additional structural complexities and conditions that are favourable for certain species to seek refuge, particularly those species of a high trophic level that are also considered of value for fishing activities. In general, the more complex a habitat is, the greater the species richness will be^89,90^, as it provides a variety of niche microhabitats/structural features suitable for particular taxa to inhabit (e.g. caves, crevices, and other interstitial spaces). Hence, the high species count observed within natural reef habitats. Fish communities on pipelines will only mimic aspects of natural reefs if they share similar habitat features favourable for refuge^91^ and while differences in habitat features remain, different fish assemblages will also be found^92,93^. Anecdotally we observed that where habitat complexity increased around small structures, such as spans, rock dumps, and concrete mattresses along the pipeline, in addition to high epifaunal growth, the abundance and number of species increased (similar to Ref.^29–34,77^). Understanding how fish utilise these small structures along the pipeline as habitat may be useful for enhancing artificial reef designs^11^. Further work is needed on nearshore pipeline systems, focusing on covariates such as pipeline features, diameter, depth, and distance from natural reefs, and a better understanding of day/night residency of fish, all of which would provide greater clarity around the ecological, social and economic value of structures associated with subsea pipelines.

## Supplementary Information

Supplementary Informations.

## Data Availability

The datasets generated during the current study are available from the corresponding author on reasonable request, via the GlobalArchive repository, https://globalarchive.org/.
