# Supplementary material for: Fish associations with shallow water subsea pipelines compared to surrounding reef and soft sediment habitats
Source: Sci Rep. 2021 Mar 18;11:6238. doi: 10.1038/s41598-021-85396-y (PMC7973564; doi:10.1038/s41598-021-85396-y)
Supplement: Supplementary file 1 — Supplementary Informations. [file 41598_2021_85396_MOESM1_ESM.pdf]

Fish associations with shallow water subsea pipelines compared to surrounding reef and soft sediment habitats

Karl D. Schramm[a], Michael J. Marnane[b], Travis S. Elsdon[b], Christopher Jones[c], Benjamin J. Saunders[a], Stephen J. Newman[a,d], and Euan S. Harvey[a*]

[a] School of Molecular and Life Sciences, Curtin University, Bentley, 6102, Western Australia, Australia.

[b] Chevron Technical Center, 250 St Georges Tce, Perth, 6000, Western Australia, Australia.

[c] Chevron Australia Pty Ltd, 250 St Georges Tce, Perth, 6000, Western Australia, Australia

[d] Western Australian Fisheries and Marine Research Laboratories, Department of Primary Industries and Regional Development, Government of Western Australia, P.O. Box 20, North Beach 6920, Western Australia, Australia

*Corresponding author: Euan S. Harvey (euan.harvey@curtin.edu.au)

Supplementary Table S1. Feeding guild of taxa

Taxa	Feeding guild	Reference
Abalistes stellatus	Invertivore	Jenkins, 2019; Kuiter and Tonozuka, 2001; Randall, 1995
Abudefduf bengalensis	Omnivore	Lieske and Myers, 1994
Abudefduf septemfasciatus	Omnivore	Hiatt and Strasburg, 1960, Lieske and Myers, 1994
Abudefduf sexfasciatus	Omnivore	Sano et al., 1984
Acanthurus grammoptilus	Herbivore	Randall, 2001
Acanthurus triostegus	Herbivore	Dominici-Arosemena and Wolff, 2006
Aluterus scriptus	Omnivore	Dominici-Arosemena and Wolff, 2006, 2005; Randall, 1967
Amphiprion clarkii	Omnivore	Miyagawa, 1989; Moe, 1992; Sano et al., 1984
Anampses caeruleopunctatus	Invertivore	Myers, 1991
Anampses geographicus	Invertivore	Sano et al., 1984
Anampses lennardi	Invertivore	Lieske and Myers, 1994; Nelson, 1994
Anampses melanurus	Invertivore	Lieske and Myers, 1994
Anampses meleagrides	Invertivore	Jenkins, 2019; Nelson, 1994
Apogonidae spp	Invertivore	Nelson, 1994
Arothron hispidus	Omnivore	Dominici-Arosemena and Wolff, 2006; Thollot, 1996
Arothron manilensis	Invertivore	Myers, 1991
Arothron mappa	Omnivore	Myers, 1991
Arothron stellatus	Invertivore	Kulbicki et al., 2005
Aspidontus taeniatus	Generalist Carnivores	Kuiter and Tonozuka, 2001; Myers, 1991
Atule mate	Invertivore	Fischer et al., 1990; Yamashita et al., 1987
Aurigequula longispina	Generalist Carnivores	Blaber, 1980; Fischer et al., 1990; Masuda and Allen, 1993
Blenniidae spp	Omnivore	Nelson, 1994
Caesio caerulaurea	Planktivore	Jenkins, 2019
Caesio cuning	Planktivore	Carpenter, 1987
Canthigaster valentini	Omnivore	Myers, 1991
Carangoides fulvoguttatus	Generalist Carnivores	Fischer et al., 1990; Gell and Whittington, 2002
Centropyge tibicen	Herbivore	Masuda and Allen, 1993; Myers, 1991
Cephalopholis boenak	Generalist Carnivores	Blaber et al., 1990, Craig et al., 2011
Cephalopholis miniata	Generalist Carnivores	Shpigel and Fishelson, 1989
Chaetodon adiergastos	Invertivore	Pyle, 2001
Chaetodon assarius	Omnivore	Steene, 1978
Chaetodon aureofasciatus	Corallivore	Pratchett, 2005
Chaetodon auriga	Omnivore	Myers, 1991
Chaetodon citrinellus	Omnivore	Harmelin-Vivien, 1989; Myers, 1991
Chaetodon lunula	Omnivore	Harmelin-Vivien, 1989; Heemstra, 1986
Chaetodon plebeius	Corallivore	Pratchett, 2005; Sano et al., 1984; Steene, 1978
Chaetodon trifascialis	Corallivore	Steene, 1978, Sano et al., 1984
Chaetodontoplus duboulayi	Invertivore	Randall et al., 1990
Cheilinus chlorourus	Invertivore	Sano et al., 1984
Cheilio inermis	Invertivore	Myers, 1999
Cheilodactylus gibbosus	Omnivore	Allen and Heemstra, 1976

Taxa	Feeding guild	Reference
Chelmon marginalis	Invertivore	Anderson et al., 1981; Lieske and Myers, 1994
Chlorurus microrhinos	Herbivore	Bacchet et al., 2005
Chlorurus sordidus	Herbivore	Sommer et al., 1996
Choerodon cauteroma	Invertivore	Randall, 1990
Choerodon cephalotes	Invertivore	Randall, 1990
Choerodon cyanodus	Invertivore	Randall, 1990
Choerodon schoenleinii	Invertivore	Randall et al., 1990
Choerodon vitta	Invertivore	Randall, 1990
Chromis fumea	Planktivore	Jan, 1997; Wantiez, 1994
Chromis viridis	Planktivore	Emslie et al., 2019, Sano et al., 1984
Chromis weberi	Planktivore	Emslie et al., 2019
Cirrhitichthys aprinus	Generalist Carnivores	Hiatt and Strasburg, 1960
Coradion chrysozonus	Invertivore	Lieske and Myers, 1994
Coris aygula	Invertivore	Sano et al., 1984; Westneat, 2001
Coris caudimacula	Invertivore	Randall, 1999
Coris pictoides	Invertivore	Jenkins, 2019
Ctenochaetus striatus	Omnivore	Axe, 1990; Sano et al., 1984; Sluka and Miller, 2001
Dascyllus aruanus	Omnivore	Jenkins, 2019; Sano et al., 1984
Dascyllus reticulatus	Herbivore	Hobson and Chess, 1978
Dascyllus trimaculatus	Omnivore	Allen, 1991
Diagramma pictum labiosum	Generalist Carnivores	Sommer et al., 1996
Diploprion bifasciatum	Piscivores	Jenkins, 2019; Paxton et al., 1989
Echeneis naucrates	Generalist Carnivores	Smith, 1997
Ecsenius bicolor	Herbivore	Jenkins, 2019
Epibulus insidiator	Generalist Carnivores	Lieske and Myers, 1994
Epinephelus bilobatus	Generalist Carnivores	Craig et al., 2011; Kulbicki et al., 2005
Epinephelus coioides	Generalist Carnivores	Kilbicki et al., 2005; Randall and Heemstra, 1991
Epinephelus fasciatus	Generalist Carnivores	Harmelin-Vivien and Bouchon, 1976; Morgans, 1982; Randall and Ben-Tuvia, 1983
Epinephelus malabaricus	Generalist Carnivores	Lieske and Myers, 1994
Epinephelus merra	Generalist Carnivores	Thollot, 1996
Epinephelus multinotatus	Generalist Carnivores	Kailola et al., 1993; Parrish, 1987
Epinephelus quoyanus	Generalist Carnivores	Craig et al., 2011; Heemstra and Randall, 1993
Epinephelus rivulatus	Generalist Carnivores	Sommer et al., 1996
Glaucosoma magnificum	Invertivore	McKay, 1997
Gnathanodon speciosus	Generalist Carnivores	Allen and Erdmann, 2012; Lieske and Myers, 1994
Gobiidae spp	Invertivore	Swainston, 2010
Gomphosus varius	Generalist Carnivores	Randall et al., 1990
Gymnothorax thrysoideus	Generalist Carnivores	Bacchet et al., 2005, Swainston, 2010
Halichoeres margaritaceus	Generalist Carnivores	Myers, 1991
Halichoeres melanochir	Invertivore	Sano et al., 1984
Halichoeres nebulosus	Invertivore	Sano et al., 1984
Halichoeres prosopeion	Invertivore	Myers, 1999; Randall et al., 1997
Hemigymnus fasciatus	Invertivore	Sano et al., 1984; Westneat, 2001
Hemigymnus melapterus	Invertivore	Myers, 1999; Westneat, 2001
Heniochus acuminatus	Planktivore	Jenkins. 2019; Masuda and Allen, 1993

Taxa	Feeding guild	Reference
Heniochus monoceros	Invertivore	Allen, 1985; Anderson and Hafiz, 1987
Hologymnosus annulatus	Generalist Carnivores	Randall et al., 1990
Hologymnosus doliatus	Generalist Carnivores	Cornic, 1987, Myers, 1991
Hoplolatilus cuniculus	Invertivore	Dooley, 1978
Labroides dimidiatus	Invertivore	Westneat, 2001, Kuiter and Tonozuka, 2001
Leptojulis cyanopleura	Planktivore	Randall, 1996
Lethrinus atkinsoni	Generalist Carnivores	Carpenter, 1997, Kilbicki et al., 2005
Lethrinus genivittatus	Generalist Carnivores	Carpenter and Allen, 1989; Kilbicki et al., 2005; Sano et al., 1984
Lethrinus laticaudis	Generalist Carnivores	Carpenter and Allen, 1989; Salini et al., 1994
Lethrinus lentjan	Generalist Carnivores	Carpenter and Allen, 1989, Kilbicki et al., 2005, Salini et al., 1994
Lethrinus nebulosus	Generalist Carnivores	Salini et al., 1994; Walker, 1978
Lethrinus punctulatus	Generalist Carnivores	Allen, 2009
Lutjanus carponotatus	Generalist Carnivores	Connell, 1998
Lutjanus fulviflamma	Generalist Carnivores	Sommer et al., 1996
Lutjanus lemniscatus	Generalist Carnivores	Allen, 1985
Lutjanus lutjanus	Generalist Carnivores	Allen, 1985; Sommer et al., 1996
Lutjanus monostigma	Generalist Carnivores	Myers, 1999
Lutjanus quinquelineatus	Generalist Carnivores	Allen, 1984
Lutjanus sebae	Generalist Carnivores	Allen, 1985
Lutjanus vitta	Generalist Carnivores	Allen, 1985; Salini et al., 1994
Macropharyngodon negrosensis	Invertivore	Jenkins, 2019
Meiacanthus grammistes	Generalist Carnivores	Myers, 1999
Monacanthus chinensis	Omnivore	Bell et al., 1978; Conacher et al., 1979
Monotaxis grandoculis	Invertivore	Carpenter and Allen, 1989; Kulbicki, et al., 2005
Mulloidichthys vanicolensis	Invertivore	Honebrink, 1990
Naso brachycentron	Herbivore	Sommer et al., 1996
Naso brevirostris	Omnivore	Choat et al., 2002; Randall, 1985
Naso lituratus	Herbivore	Sluka and Miller, 2001
Naso unicornis	Herbivore	Choat et al., 2002; Jenkins, 2019; Tinker, 1978
Nebrius ferrugineus	Generalist Carnivores	Compagno, 1984; Cortés, 1999; Myers, 1999
Nelusetta ayraud	Generalist Carnivores	Lindholm, 1984
Nemipterus furcosus	Generalist Carnivores	Salini et al., 1994, Russell, 1990
Neoglyphidodon melas	Corallivore	Broad, 2003; Jenkins, 2019; Myer, 1991
Neoglyphidodon nigroris	Omnivore	Allen, 1991
Neopomacentrus azysron	Planktivore	Hammer et al., 1988
Neopomacentrus aktites	Planktivore	Allen et al., 2017
Neotrygon australiae	Generalist Carnivores	Swainston, 2010
Ostorhinchus angustatus	Invertivore	Myers, 1991
Ostorhinchus semilineatus	Invertivore	Horinouchi and Sano, 2000
Ostracion cubicus	Omnivore	Cornic, 1987; Myers, 1991, 1999
Oxymonacanthus longirostris	Corallivore	Barlow, 1987
Parachaetodon ocellatus	Invertivore	Allen, 2006; Kuiter and Tonozuka, 2001
Paramonacanthus choirocephalus	Invertivore	Allen and Erdmann, 2012; Kuiter and Tonozuka, 2001
Parapercis nebulosa	Invertivore	Allen and Swainston, 1988

Taxa	Feeding guild	Reference
Paraplotosus butleri	Invertivore	Allen, 1998
Parupeneus barberinoides	Invertivore	Myers, 1991
Parupeneus heptacanthus	Invertivore	Kilbicki et al., 2005
Parupeneus indicus	Generalist Carnivores	Jenkins, 2019; Randall, 2004
Parupeneus spilurus	Invertivore	Sano et al., 1984, Jenkins, 2019
Pateobatis jenkinsii	Generalist Carnivores	Last and Compagno, 1999
Pempheris analis	Planktivore	Randall et al., 1990
Pempheris oualensis	Generalist Carnivores	Hiatt and Strasburg, 1960
Pempheris schwenkii	Planktivore	Randall et al., 1990
Pentapodus emeryii	Generalist Carnivores	Jenkins, 2019; Russell, 1990
Pentapodus porosus	Generalist Carnivores	Russell, 1990
Pentapodus vitta	Generalist Carnivores	Russell, 1990
Petroscirtes breviceps	Omnivore	Sano et al., 1984
Plagiotremus tapeinosoma	Generalist Carnivores	Jenkins, 2019; Sano et al., 1984
Platax batavianus	Generalist Carnivores	Lieske and Myers, 1994
Plectorhinchus multivittatus	Invertivore	Jenkins, 2019
Plectorhinchus polytaenia	Invertivore	Allen and Erdmann, 2012; Lieske and Myers, 1994
Plectropomus spp	Piscivores	Kailola et al., 1993; Kingsford, 1992
Pomacanthus imperator	Invertivore	Anderson and Hafiz, 1987
Pomacanthus semicirculatus	Omnivore	Sommer et al., 1996
Pomacanthus sexstriatus	Omnivore	Allen et al., 1998, Jenkins, 2019
Pomacentrus amboinensis	Omnivore	Allen, 1991; Sano et al., 1984
Pomacentrus coelestis	Omnivore	Hobson and Chess, 1978; Jenkins, 2019
Pomacentrus limosus	Omnivore	Evans et al., 2014
Pomacentrus milleri	Omnivore	Allen, 1991
Pomacentrus moluccensis	Omnivore	Allen, 1991,
Pomacentrus nagasakiensis	Omnivore	Allen,1975, 1991
Pomacentrus vaiuli	Omnivore	Jenkins, 2019, Myers, 1991
Psammoperca datnioides	Generalist Carnivores	Jenkins, 2019
Pseudodax moluccanus	Omnivore	Westneat, 2001
Ptereleotris evides	Planktivore	Randall and Hoese, 1985; Sano et al., 1984
Ptereleotris microlepis	Planktivore	Bacchet et al., 2005
Pterocaesio chrysozona	Planktivore	Carpenter, 1987,1988; Bellwood, 1988
Pterocaesio lativittata	Planktivore	Carpenter, 1988
Pterocaesio tile	Planktivore	Bellwood, 1988; Carpenter, 1988
Pterois volitans	Generalist Carnivores	Myer, 1991; Sano et al., 1984
Sargocentron rubrum	Generalist Carnivores	Göthel, 1992; Randall et al., 1990
Scarus forsteni	Herbivore	Bacchet et al., 2005
Scarus ghobban	Herbivore	Humann and Deloach, 1993
Scarus prasiognathos	Herbivore	Bruce and Randall, 1984
Scarus psittacus	Herbivore	Bruce and Randall, 1984
Scarus rivulatus	Herbivore	Schroeder, 1980
Scarus schlegeli	Herbivore	Bacchet et al., 2005
Scolopsis affinis	Generalist Carnivores	Jenkinsn 2019; Mequila and Campos, 2007
Scolopsis bilineata	Generalist Carnivores	Russell, 1990
Scolopsis monogramma	Generalist Carnivores	Jenkins, 2019; Russell, 1990; Salini et al., 1994
Scomberoides lysan	Generalist Carnivores	Fischer et al., 1990

**Taxa**	**Feeding guild**	**Reference**
Scomberomorus spp	Generalist Carnivores	Bachok et al., 2004; Jenkins, 2019
Selaroides leptolepis	Generalist Carnivores	Paxton et al., 1989; Yamashita et al., 1987
Siganus argenteus	Herbivore	Sommer et al., 1996
Siganus canaliculatus	Herbivore	Cuihong et al., 2014; Soh, 1976
Siganus doliatus	Herbivore	Bennett and Bellwood, 2011; Cheal et al., 2010
Siganus fuscescens	Herbivore	Lieske and Myers, 1994; Pillans et al., 2004
Siganus lineatus	Herbivore	Fox et al., 2009; Thollot, 1996
Siganus punctatus	Herbivore	Rhodes et al., 2017; Woodland, 1997
Siganus trispilos	Herbivore	Woodland and Allen, 1977
Sphyraena jello	Piscivores	Bachok et al., 2004; Jenkins, 2019
Sphyraena obtusata	Piscivores	Kilbicki et al., 2005; Thollot, 1996
Stegastes fasciolatus	Herbivore	Jenkins, 2019
Stegastes nigricans	Herbivore	Hata and Kato, 2004
Stegastes obreptus	Herbivore	Allen and Emery, 1985; Hata and Kato, 2004
Stethojulis bandanensis	Invertivore	Sano et al., 1984
Stethojulis interrupta	Invertivore	Randall, 2000
Stethojulis strigiventer	Invertivore	Gell and Whittington, 2002; Randall, 2000, Sano et al., 1984
Sufflamen chrysopterum	Invertivore	Myers, 1991
Symphorus nematophorus	Piscivores	Allen, 1985; Kilbicki et al., 2005
Synodus variegatus	Generalist Carnivores	Bacchet et al., 2005
Thalassoma amblycephalum	Planktivore	Jenkins, 2019
Thalassoma hardwicke	Generalist Carnivores	Jenkins, 2019; Myers, 1991; Randall et al., 1990
Thalassoma lunare	Invertivore	Westneat, 2001
Thalassoma lutescens	Invertivore	Sano et al., 1984
Torquigener whitleyi	Invertivore	Sainsbury et al., 1985
Triaenodon obesus	Generalist Carnivores	Compagno, 1984
Upeneus tragula	Invertivore	Jenkins, 2019; Kilbicki et al., 2005
Yongeichthys nebulosus	Invertivore	Allen, 1997
Zanclus cornutus	Invertivore	Anderson and Hafiz, 1987; Dominici-Arosemena and Wolff, 2006; Hobson, 1975
Zebrasoma scopas	Herbivore	Guiasu and Winterbottom, 1998

Supplementary Table S2. The value of commercial fish (AUD/kg) based on the mean wet weight market value for commercial species for 2017/2018 (Gaughan et al., 2019). C = Commercial Fisheries; MC = Minor Commercial Fisheries; R = Recreational Fisheries; AQM = Aquarium Fisheries; AQC = Aquaculture Fisheries; AF = Artisanal Fisheries.

Row Labels	Common name	Catch value ($AUD/kg)	Target Status
Carangoides fulvoguttatus	Yellowspotted trevally	1.70	C/R
Cephalopholis boenak	Brownbarred Rockcod	7.15	C/R/AQM
Cephalopholis miniata	Coral rockcod	9.84	C/R
Choerodon cauteroma	Bluespotted tuskfish	6.80	C/R
Choerodon cephalotes	Purple tuskfish	6.80	C/R
Choerodon cyanodus	Blue tuskfish	6.80	C/R/AQM
Choerodon schoenleinii	Blackspot tuskfish	6.80	C/R/AQM
Diagramma pictum labiosum	Painted sweetlips	4.95	C/R
Epinephelus bilobatus	Frostback rockcod	5.85	C/R
Epinephelus coioides	Goldspotted rockcod	6.77	C/R/AQC
Epinephelus fasciatus	Blacktip rockcod	5.47	C/R
Epinephelus malabaricus	Blackspotted rockcod	6.99	C/R
Epinephelus merra	Birdwire rockcod	9.84	C/R/SF
Epinephelus multinotatus	Rankin cod	8.08	C/R
Epinephelus rivulatus	Chinaman rockcod	5.47	-
Glaucosoma magnificum	Threadfin pearl perch	7.03	C/R
Gnathanodon speciosus	Golden trevally	3.08	C/R/AQM
Lethrinus atkinsoni	Yellowtail emperor	4.42	C
Lethrinus laticaudis	Grass emperor	6.76	C/R
Lethrinus lentjan	Redspot emperor	4.95	C/R
Lethrinus nebulosus	Spangled emperor	6.00	C/R
Lethrinus punctulatus	Bluespotted emperor	4.28	C/R
Lutjanus carponotatus	Stripey snapper	3.82	C/R
Lutjanus lemniscatus	Darktail snapper	5.39	C/R
Lutjanus quinquelineatus	Fiveline snapper	3.82	C/R
Lutjanus sebae	Red emperor	11.31	C/R/AQM
Lutjanus vitta	Brownstripe snapper	3.82	C/R
Monotaxis grandoculis	Bigeye seabream	5.22	C/R/AF
Nelusetta ayraud	Chinaman-leatherjacket	4.35	C/R
Plectropomus spp	Coral trout	14.91	C/R/AQC
Psammoperca datnioides	Black sand bass	7.86	C/R
Scolopsis monogramma	Rainbow Monocle bream	1.89	C
Scomberoides lysan	Doublespotted queenfish	5.10	MC/R
Scomberomorus spp	Mackerel	9.54	C/R
Siganus fuscescens	Black rabbitfish	3.96	C/AQC
Siganus lineatus	Goldlined rabbitfish	3.96	C
Sphyraena jello	Pickheadle barracuda	3.99	C/R
Sphyraena obtusata	Obtuse barracuda	3.99	R
Symphorus nematophorus	Chinamanfish	5.48	C/R
